# Belief in a just world and fair behavior among clinical nurses: a moderated mediation model of empathy and observer justice sensitivity

**DOI:** 10.1186/s12912-024-02140-3

**Published:** 2024-07-15

**Authors:** Youjuan Hong, Bo Zhu, Caimei Chen, Meichai Qiu, Liting Liu

**Affiliations:** 1 School of Nursing, Fujian Medical University, Fuzhou, China; 2 School of Marxism, Fujian Medical University, Fuzhou, China; 3 Critical Care Department, Longyan People’s Hospital, Longyan, Fujian China; 4 Center for information Management, Fujian Provincial Hospital, Fuzhou, Fujian China; 5 School of Management Studies, Shanghai University of Engineering Science, Shanghai, China

**Keywords:** Belief in a just world, Fair behavior, Empathy, Observer justice sensitivity, Mediation, Moderation

## Abstract

**Background:**

Exploration of the relationship between nursing staffs’ justice in belief world and fair behavior is important to promote equity and access to health services in health organizations, as well as to enhance the quality of care. In order to further dissect the influencing factors of fair behavior among clinical nurses, the current study aims to investigate how belief in a just world influences the fair behavior among nurses. Based on the belief in a just world theory, the empathy-altruism theory and the protective-protective model, the current study aimed to provide a deeper understanding of the effect of belief in a just world on fair behavior by investigating the mediating role of empathy and the moderating role of observer justice sensitivity.

**Method:**

This was a cross-sectional study. 571 registered clinical nurses were included from five hospitals in Fuzhou through a convenience sampling method. Measurements included Chinese translations of belief in a just world scale, empathy scale, observer justice sensitivity scale, fair behavior scale. SPSS 22.0 was used to describe descriptive statistics and the variables’ Pearson correlation coefficient. SPSS PROCESS macro Model 4 and model 14 were used to examine the mediation and the moderation between the relationship of belief in a just world and fairness behavior.

**Result:**

The results shower that fairness behavior was positively correlated with one’s belief in a just world (*r* = 0.26, *p* < 0.01); (2)empathy mediated the relationship between belief in a just world and fair behavior. The mediation model explains 20.83%; (3) Observer justice sensitivity moderated the relationship between empathy and fair behavior.

**Conclusions:**

Belief in a just world, empathy, and observer justice sensitivity were motivations for nurses’ fair behavior. Nursing administrators should focus on cultivating nurses’ belief in a just world, their empathy abilities, and positive qualities of justice sensitivity to enhance fair behavior in a healthcare setting.

## Introduction

Social justice is a fundamental concern in all social organizations and is deeply rooted in human nature [1]. According to behavioral ethics theorists, ethical behavior must include a commitment to treating others fairly [2]. In recent decades, organizational fairness has been a hot topic, and a series of influences of justice have been obtained [3]. The reaction to perceived fairness or unfairness was also found in a healthcare setting. Previous research found that perceived justice will affect patients’ reactions to their clinicians and to the health system in general [4, 5]. For instance, patients’ perceived justice predicts their attitude and behavior in the health care professional [6]. Specifically, fairness can increase patients’ satisfaction and improve the physician-patient relationship [7, 8].

Despite the considerable knowledge gained regarding the impact of fairness on outcomes [9–11], it is important to acknowledge that the current body of literature has certain limitations. First, health organizational justice research traditionally focuses on patients’ reactions to fairness [6]. Little attention has been given to why nurses behave fairly or unfairly in the first place. Nor do we know much about the factors that influence nurses’ fair behavior. Fair behavior is an important part of nurses’ professional quality and humanistic spirit. Exploring what drives the fair behavior of nurses is important in understanding fairness in health organizations. In addition, researchers have not adequately identified the inner mechanisms of the relationship between influence factors and fair behavior (i.e., mediation, moderation). Belief in a just world (BJW) refers to an individual’s equitable disposition towards their surroundings, potentially influencing how inclined they are to behave fairly [12, 13]. Additionally, fair behavior is challenging to achieve if the person lacks empathy [14]. Empathy is also a driver of fair behavior. In addition, research also found that personality factors influence fair behavior positively [15, 16]. Observer justice sensitivity as a personality trait that influences reactions to injustice may moderate the relationship between BJW and fair behavior.

Therefore, the current research addresses these concerns to contribute new knowledge to the field. Our focus here is on answering two questions related to nurses’ ethical conduct: (1) what is the process by which BJW and empathy affects fair behavior and (2) are personality traits like observer justice sensitivity moderating the relationship between BJW and fair behavior? Knowing the effect of intra-individual factors on medical staff’s fair behaviors may be helpful to promote the development of their fair behaviors, and cultivating exceptional medical professionals, enhancing communication skills, and strengthening doctor-patient bonds are all areas of great importance and value that can be advanced through theoretical study and clinical practice.

## Background

### BJW and fair behavior

The concept of belief in a just world refers to the extent to which individuals hold the conviction that the world operates fairly and harmoniously and that they will be treated equitably by others [17]. The belief in a just world theory is based on the assumption that our world is predictable, fundamentally just, and governed by a certain order [18]. It is the individual’s explanation of their physical surroundings and the spiritual pursuit of equity [19]. Belief in a just world as a justice motivation compels individuals to behave justly; high believers in a just world will be motivated to achieve their personal goals by just means [20]. The more individuals believe in a just world, the more they will behave justly, trust in their future, and see events in their life as more just [17–21]. According to the just world hypotheses, individuals who believed strongly in a just world tended to engage in behavior aimed at restoring justice if an injustice had been committed [18]. In this regard, it seems reasonable to assume that nurses who believe the world is a just place might act toward patients fairly, regardless of social class, economic background, or severity of the disease of patients. Therefore, consistent with the belief in a just world theory, the current study aimed to explore the relationship between belief in a just world and fair behavior among nurses.

### Empathy as a mediator

Empathy refers to the ability that facilitates the understanding of the emotions of others (i.e., cognitive empathy) and experience of an emotional reaction coherent with the other person’s affective state (i.e., affective empathy) [22]. Previous research has reported that BJW was positively associated with empathy [23]. Nurses with a higher level of BJW are inclined to believe that they will receive fair and equitable treatment. Additionally, these nurses anticipate their efforts and understanding will be duly acknowledged and reciprocated. Consequently, they are more inclined to adopt a patient-centered perspective, leading to heightened levels of empathy [13]. Adversely, nurses who possess a diminished level of belief in a just world (BJW) are inclined to perceive the world with a lens of “bias”, “unfairness”, and “self-orientation”. Consequently, these nurses encounter challenges in empathizing with patients and comprehending their emotional encounters.

In addition, empathy is considered a driving motivation of moral behavior and justice [24]. It arises from the motivation to alleviate the suffering of others, is seen as a moral emotion, and often leads to prosocial behavior [25, 26]. According to the empathy-altruism hypothesis, when others are in trouble, bystanders will experience emotions such as compassion directed towards the victim, and the stronger one’s empathy, the stronger that individual’s motivation to help others solve their difficulties [27]. Thus, empathy is an important emotional factor that triggers third-party punishment, which plays an important role in fairness norm enforcement [28]. Nurses with low empathy may fail to comfort patients in distress, and their fair behavior may be tempered by the vicarious emotional experience and/or comprehension of the emotional states of patients [29]. Therefore, nurses with high BJW have higher empathy, then increased fair behavior. Accordingly, we propose that empathy functions as a mediator in the relationship between belief in a just world and fair behavior. Specifically, belief in a just world was assumed to be positively associated empathy, and high empathy in turn is assumed to be associated with higher fair behavior.

### The moderation role of observer justice sensitivity

The mediation effect of BJW on fair behavior may be moderated by personality factors. Previous studies found that justice sensitivity reliably predicts justice behavior [30, 31]. Justice sensitivity entails four distinct dimensions: victim sensitivity, observer sensitivity, beneficiary sensitivity, and perpetrator sensitivity [32]. Prosocial behavior is associated differently with each factor [30]. For instance, individuals with higher observer justice sensitivity frequently perceive the unjust treatment of others without being involved, react with indignation, and strive for compensating the victim and/or punishing the perpetrator [33]. Specifically, individual differences in observer justice sensitivity positively correlate with compensatory behaviors toward those treated unfairly [34]. Apparently, for nurses high in observer justice sensitivity, perceiving injustice provides a strong motivation to act in order to avoid injustice or restore justice. In addition, observer justice sensitivity was found to positively predict the level of compassion for the trapped [35]. The protective-protective model indicated that a protective factor (observer justice sensitivity) enhances the effect of another protective factor (empathy) in producing an outcome [36]. Nurses with higher observer justice sensitivity will act more fairly when utilizing empathy. The higher the individual’s empathy response, the more compensatory behaviors are to the excluded [37]. In Pfattheicher’ s study [38], third-party observers with high empathy reported more punishments for unfair offers in order to maintain justice. Therefore, observer justice sensitivity may play a moderating role in the relationship between empathy and fair behavior; that is, a higher observer justice sensitivity will strengthen the effect of empathy on fair behavior.

### The present study: aims and hypotheses

**Fig. 1:**
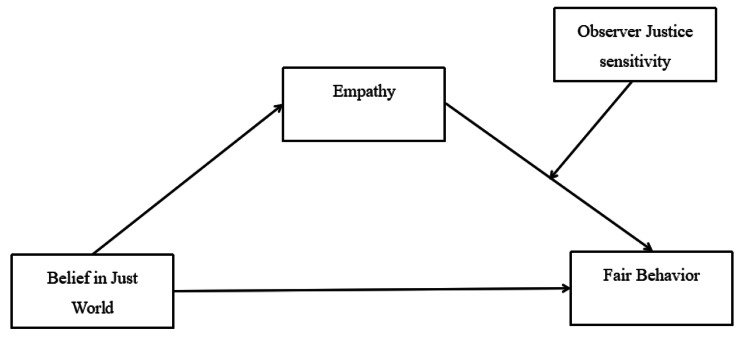
The hypothesized model

To sum up, drawing on the belief in a just world theory, the empathy-altruism theory and the protective-protective model, this study developed a moderated mediation model to investigate the foundational processes through which belief in a just world (BJW) predicts nurse’ fair behavior to provide ideas for increasing fair behavior for nurses in a healthcare setting. The results will contribute to the current literature by extending our understanding of the mechanism that connects belief in a just world and fair behavior. The hypothesized moderated mediation model is presented in Fig. 1, comprising the following three hypotheses:

#### Hypothesis 1

Belief in a just world is positively correlated with fair behavior among nurses.

#### Hypothesis 2

Empathy plays the mediation role of the relationship between belief in a just world and fair behavior among nurses.

#### Hypothesis 3

Observer justice sensitivity plays a moderation role in the relationship between empathy and fair behavior among nurses.

## Methods

### Participants and procedure

A total of 571 clinical nurses consisting of 537 females (94.04%) and 34 males (5.96%) participated in the study. The participants were recruited from five hospitals located in a Southeast city in China, including two tertiary hospitals (a large-scale general hospital with over 501 beds) and three secondary hospitals (regional hospitals with bed capacities ranging from 101 to 500).

We used convenience sampling to collect data on the Wenjuanxing online platform from 10 July to 10 September 2022 in China. The inclusion criteria were full-time nurses who worked for more than one year and voluntarily participated in the study, and the departments included are inpatient medical, surgical, and critical care units. The departments comprised inpatient medical, surgical, and critical care units. Finally, a total data of 623 answers were collected, of which 571 were valid, 31 insincerely answered questionnaires, and 21 missing data questionnaires were deleted, and the effective response rate was 91.65%. The sample size should be greater than 10 times the number of observed variables [39]. Therefore, a sample size of 571 met the requirement for further analysis.The participants’ ages ranged from 25 to 46 years (M = 33.59, SD = 5.38). The largest group was between 31 and 39 years old (51.5%), followed by those below 30 years old (35.4%), those above 40 yeas old (13.1%). The majority of participants had a bachelor’s degree (*N* = 480, 84.1%), and 87 nurses (7.6%) had a associate degree, 4 (0.7%) nurses had a master’s degree. Permission to implement the study was approved by the corresponding author’s affiliation ethics committee. The details are shown in Table 1.

**Table 1:** Demographic variables of nurses (*N* = 571)

	Frequency (%)	t/F
**Age**		F = 1.94, *P* > 0.05
Below 30	202(35.4%)	3.42 ± 0.57
31–39	294(51.5%)	3.36 ± 0.67
Above 40	75(13.1%)	3.25 ± 0.79
**Gender**		*t =* 0.07, *P* > 0.05
Male	34(6.0%)	3.37 ± 0.66
Female	537(94.0%)	3.36 ± 0.52
**Marital status**		F = 0.54, *P* > 0.05
Single	98(17.2%)	3.38 ± 0.62
Married	467(81.8%)	3.37 ± 0.66
Divorced	6(1.1%)	3.09 ± 0.65
**Level of education**		F = 0.56, *P* > 0.05
Diploma degree	87(15.2%)	3.43 ± 0.65
Bachelor’s degree	480(84.1%)	3.35 ± 0.66
Master’s degree	4(0.7%)	3.41 ± 0.87
**Years of work experience**		F = 4.01, *P* < 0.05
Less than 5 years	84(14.7%)	3.42 ± 0.58
6–15 years	351(61.5%)	3.42 ± 0.63
16–25 years	97(17.0%)	3.22 ± 0.67
More than 15 years	39(6.8%)	3.16 ± 0.83

### Measurement

#### Belief in a just world

Belief in a just world was measured by the Personal Belief in a Just World Scale [40]. This scale valued the belief that events in one’s life are just. This scale has six items rated by 5 Likert points (1 = totally disagree, 6 = totally agree). An example item is as follows: “I am convinced that in the long run, people will be compensated for injustices”. The higher the total score, the higher one’s belief in a just world. In the original study, the scale demonstrated good internal consistency (α = 0.82). The Cronbach’s alpha for this scale in the present study was 0.85.

#### Empathy

Empathy was assessed using the Empathy Scale, developed by Vossen [41]. Eight questions comprise the scale, which measures two types of empathy: cognitive (I can often understand how people are feeling even before they tell me) and emotional (When my friend is sad, I become sad too). The scale is assessed using a Likert scale with five points, where a score of 1 represents “never” and 5 represents “always”. A higher score on this scale indicates a greater level of empathy. In the original study, the subscales exhibited internal consistency (cognitive empathy α = 0.86, emotional empathy α = 0.75). In the present study, the cognitive and emotional empathy dimensions exhibited Cronbach’s alpha coefficients of 0.78 and 0.77.

#### Observer Justice Sensitivity

Observer justice sensitivity was assessed using the Chinese version of the observer justice sensitivity Scale [33]. The scale consists of 10 questions, each scored on a six-point scale from 1 (strongly disagree) to 6 (strongly agree). An example of items are the following: “I get upset when others are treated worse”. In the original study, the scale demonstrated good internal consistency (α = 0.88). The higher the participant’s score on this scale, the more sensitive they are to issues of observer justice. Cronbach’s alpha coefficient for the scale utilized in this study was determined to be 0.85.

#### Fairness behavior

Fairness behavior was measured by the Fairness Behavior Scale [42]. This scale has 12 items rated by 5 Likert points (1 = totally disagree, 5 = totally agree). An example item is as follows: “I am equally patient with all my patients”. More fair behavior is demonstrated by higher scores. In the original study, the scale demonstrated good internal consistency (α = 0.90). The Cronbach’s alpha coefficient for the scale utilized in the current study yielded a value of 0.92.

### Ethical considerations

Ethical approval was granted by the Ethics Committee of the Fujian Medical University. We obtained the informed consent from participants and informed the participants about the purpose of the study. Participants were assured of anonymity and confidentiality, they could withdraw from the study at any time.

### Data analysis

IBM SPSS Statistics 22.0 and SPSS PROCESS macro were used to analyze data in the study. SPSS 22.0 was used to describe descriptive statistics and the variables’ Pearson correlation coefficient. Simple mediation was analyzed utilizing Model 4 of the Process Macro, which elucidates the direct impact of an independent variable on an outcome variable with one or more mediators [43]. In this study, belief in a just world served as the predictor, fair behavior as the outcome variable, and empathy as the mediator. Furthermore, moderated mediation was conducted employing Model 14 of the Process Macro. This model allowed for the investigation of the mediated relationship between belief in a just world and job fair behavior via empathy, considering varying levels of observer justice sensitivity. In the study, quantitative variables were characterized using descriptive statistics, including the mean and standard deviation (SD) for normally distributed data, or the median (Mdn) and interquartile range (IQR; Q3) for non-normally distributed data. Normality assumptions were verified by the Shapiro-Wilk test in the present study.

### Common method variance test

Harman’s single-factor test was used to assess the possibility of common method bias; no common method variance was detected (18.44% interpretation rate for the first factor < 40%) [44]. Thus, no common method variance was found in the current study. Furthermore, prior to investigating the hypothesized relationships in our model, we conducted a collinearity assessment. The results indicated that none of the variance inflation factor (VIF) values exceeded the threshold of 3.3 (ranging from 1.07 to 1.27). Hence, it can be inferred that common method variance does not pose a concern in the dataset of this study.

## Results

### Descriptive statistics and correlations

Table 2 presents the variables’ correlation relationship. Results showed that BJW was positively correlated to empathy (*r* = 0.19, *p* < 0.01), observer justice sensitivity (*r* = 0.25, *p* < 0.01) and fairness behavior(*r* = 0.26, *p* < 0.01). The results also showed that empathy was positively correlated with fairness behavior (*r* = 0.24, *p* < 0.01). Moreover, observer justice sensitivity was positively correlated with empathy (*r* = 0.43, *p* < 0.01) and fairness behavior (*r =* 0.24, *p* < 0.01).

**Table 2:** Descriptive statistics

	M	SD	1	2	3	4
1. BJW	4.26	0.83	-			
2. Empathy	3.28	0.43	0.19^***^	1		
3. Observer justice sensitivity	4.17	0.65	0.25^***^	0.43^***^	1	
4. Fairness behavior	3.37	0.66	0.26^***^	0.24^***^	0.24^***^	1

Note: M = mean; SD = standard deviation. ^***^p < 0.001

### Mediation model tests

Gender, age, level of education, marital status and years of work experience were controlled as covariables. After controlling for gender and age, level of education, marital status, years of work experience, BJW was found to significantly predict fairness behavior (Model 1: *β* = 0.24, *p* < 0.001) and empathy (Model 2: *β* = 0.20, *p* < 0.001). Moreover, empathy significantly correlated fairness behavior (Model 3: *β* = 0.19, *p* < 0.001), and the direct association between belief in a just world and fairness behavior remained significant (Model 3: *β* = 0.19, *p* < 0.001). The bias-corrected percentile bootstrap analyses showed that empathy partially mediated the relationship between belief in a just world and fairness behavior (indirect effect = 0.05, Boot SE = 0.02, 95% CI = [0.02, 0.10]). The mediation model explains 20.83%. Therefore, H2 was supported (see Table 3).

**Table 3:** Testing for mediation effect

Predictors		Model 1(Fairness behavior)			Model 2(Empathy)			Model 3(Fairness behavior)	
		*β*	*t*		*β*	*t*		*β*	*t*
Gender		0.16	1.10		-0.21	-1.31		0.14	0.98
Age		0.01	1.68		0.01	1.68		-0.01	-1.98
Level of Education		-0.07	-0.86		-0.13	-1.92		-0.15	-1.70
Marital status		0.02	0.52		-0.01	-0.12		0.04	0.49
Years of work experience		-0.09	-1.06		-0.16	-1.69		-0.07	-0.90
BJW		0.24***	5.07		0.20***	3.50		0.19***	4.87
Empathy								0.19***	5.10
*R* ^*2*^		0.07			0.04			0.11	
*F*		39.97***			8.67***			17.71***	

**p < 0.01

***p < 0.001

### The moderation of observer justice sensitivity

Gender, age, level of education, marital status, and years of work experience were controlled as covariates. PROCESS Macro Model 14 assumes that the second half of the mediation model is moderated, consistent with the theoretical model of this study. In this model, belief in a just world served as the independent variable (X), fair behavior as the dependent variable (Y), empathy as the mediator (M), and the level of observer justice sensitivity as the moderator (W). As shown in Table 4, belief in a just world had a positively significant predictive effect on fair behavior (*β* = 0.17, *p* < 0.001); the interaction effect of empathy and observer justice sensitivity on fair behavior was significant (*ß* = 0.11, *p* < 0.05), and empathy had a positive predictive effect on fair behavior (*ß* = 0.19, *p* < 0.01). The results indicated that the predictive effect of empathy on fair behavior increases with an increase in individuals’ observer justice sensitivity. Therefore, H3 was supported (see Table 4).

**Table 4:** Coefficients for the tested moderated mediation model

Predictors		Model 1(Empathy)				Model 2(Fairness behavior)		
		*β*	*SE*	*t*		*β*	*SE*	*t*
Gender		0.21	0.16	1.31		0.16	0.14	1.19
Age		0.01	0.01	1.68		-0.01	0.01	-1.55
Level of Education		-0.19	0.10	-1.92		-0.15	0.09	-1.76
Marital status		-0.01	0.11	-0.12		0.05	0.10	0.55
Years of work experience		-0.16	0.09	-1.69		-0.04	0.09	-0.47
Belief in a just world		0.20	0.04	4.75		0.17***	0.04	4.36
Empathy						0.19**	0.04	4.34
Observer Justice Sensitivity						0.14*	0.04	2.91
Empathy × Observer Justice Sensitivity						0.11*	0.05	2.84
*R* ^*2*^		0.04				0.14		
*F*		8.67***				14.73***		

Note: × represents the interaction item of Empathy × Observer Justice Sensitivity. Observer Justice Sensitivity was entered as a continuous variable for Empathy × Observer Justice Sensitivity

**p < 0.01, ***p < 0.001

**Fig. 2:**
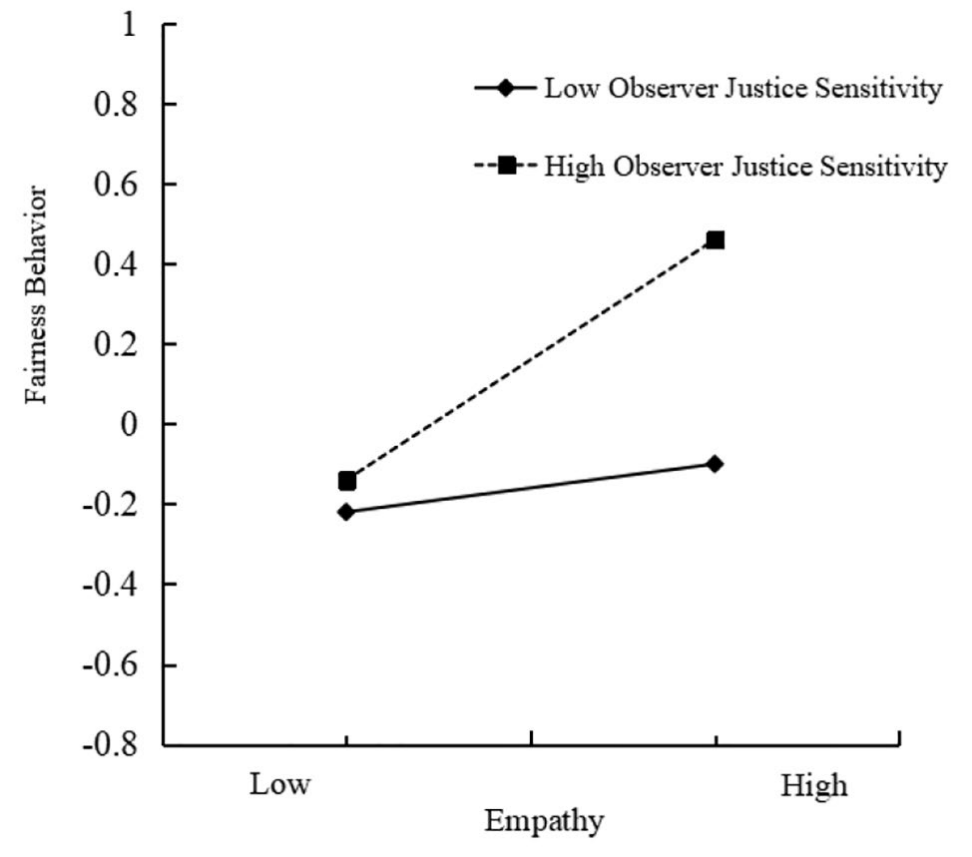
Interaction between empathy and observer justice sensitivity

Additionally, a simple slope analysis was conducted to analyze the moderating effect of observer justice sensitivity. The observer justice sensitivity score higher than M + SD represented the high group, while the score lower than M - SD represented the low group (see Fig. 2). In both high and low observer justice sensitivity groups, empathy exhibited a positive effect on fairness behavior. In the high observer justice sensitivity group, the effect of empathy on fairness behavior (*β* = 0.05, *p* < 0.001) was stronger than that in the low group (*β* = 0.01, *p* < 0.001). As empathy increased, fairness behavior showed a more pronounced increase for the high observer justice sensitivity group compared to the low observer justice sensitivity group. Specifically, the predictive effect of empathy on fair behavior increased as individuals’ observer justice sensitivity increased.

## Discussion

Based on the just world hypothesis, empathy-altruism theory, and the protective-proactive model, this study supports the motivating effects of BJW and empathy on nurses’ acts of fairness, as well as a moderated mediation model in which observer justice sensitivity acts as a personality trait, influencing the effects of empathy on nurses’ fair behavior. Specifically, this study illustrates the relationship between nurses’ belief in a just world (BJW) and their likelihood to engage in fair behaviors. It also found that the mediating role of empathy in explaining why nurses with higher BJW are more likely to exhibit fair behaviors. Additionally, the study found that observer justice sensitivity moderated the relationship between empathy and fair behavior among nurses. This is the first empirical study to explore fair behavior in a healthcare setting from the perspective of fair actors. The findings of this study contribute to a greater understanding of the association between BJW and fair conduct within a healthcare environment. Additionally, they broaden the scope of research in fairness and offer valuable insights for promoting equitable behavior within healthcare organizations.

The result showed that nurses’ BJW positively correlated with fair behavior. The results coincide with the just world hypotheses. The results confirmed the effect of individuals’ BJW on actor’s fair behavior in a healthcare setting. When nurses believe in a just world in which “everyone receives what they deserve and deserves what they receive” [45], this belief serves an adaptive social function and motivation to defend nurses’ attitudes toward a just world. In the clinical nursing process, a higher level of belief in a just world among nurses correlates with a greater likelihood of uniformly applying nursing attitudes, such as demonstrating an equivalent level of respect, to diverse patients, including those who are disadvantaged. This includes offering them consistent treatment information and medical resources, regardless of patients’ economic status, age, or appearance. Conversely, nurses with lower levels of belief in a just world are more susceptible to the influence of factors such as patients’ socioeconomic status and age. This susceptibility may result in biases against disadvantaged patients, such as those with lower socioeconomic status or elderly patients, leading to potentially unfair behaviors such as extended wait times and less friendly attitudes towards these individuals. The motive function of BJW on fair behavior provides a key to understanding why nurses behave justly. Our study is the first, to our knowledge, to illustrate the importance of justice belief in fair behavior in health organizations.

The current study found that BJW not only directly and positively predicted nurses’ fair behavior, but also affected their fair acting through their empathy. That is, empathy plays a mediating role between BJW and fair behavior in nurses, which is consistent with the empathy-altruism hypothesis [46]. The belief in a just world as one’s spiritual pursuit of fairness and justice enhances nurses’ fair behavior via the ability of empathy. Specifically, nurses with higher beliefs about a just world may experience increased understanding and empathy toward the distress, anxiety, and other emotions of disadvantaged patients. This heightened empathy can lead to the development of a greater capacity for empathy and activation of intrinsic altruistic motivation [47]. Consequently, nurses are more likely to reduce biases and differential treatment towards vulnerable patients, enabling them to impartially care for each patient, allocate medical resources fairly based on patients’ needs, and provide uniformly high-quality nursing services [48, 49], rather than offering disparate care services based on factors such as patient age, gender, race, religion, or economic status.

The results also revealed that the personality factor (observer justice sensitivity) moderated the relationship between nurses’ empathy and fair behavior. The impact of empathy on fair behavior varied with the level of observer justice sensitivity, which can be explained by the protect-protect model. Specifically, observer justice sensitivity, as a protective factor, enhances the protective effect of empathy on fair behavior among nurses. This study provides empirical evidence for the first time on the influence of observer justice sensitivity on the relationship between nurses’ belief in a just world (BJW) and fair behavior. Observer justice sensitivity provides strong intrinsic motivation for nurses’ fairness behaviors, and nurses with high observer justice sensitivity tend to focus on disadvantaged patients, care about the negative feelings of disadvantaged patients, and are willing to provide them with care on equal terms. Additionally, observer justice sensitivity interacts with empathy to promote nurses’ fair behavior. Nurses who are more indignant about unfair events are more likely to act in a just, fair, and open manner when providing nursing services to patients. They adhere to principles of justice, treat everyone equally, and do not discriminate against anyone, providing each patient with equal attention and care. Specifically, individuals with high levels of observer justice sensitivity experience a more significant enhancement in the facilitating role of empathy on fair behavior compared to those with low levels of observer justice sensitivity.

### Implication

The findings of this study have significant practical implications for enhancing the atmosphere of justice and the quality of care in healthcare settings. Belief in a Just World (BJW) was identified as a crucial predictor of justice-related behavior among medical staff. Therefore, promoting equitable care for patients from diverse backgrounds among nurses can be achieved by increasing their levels of belief in fairness toward the world. Interventions should be implemented to enhance nurses’ understanding and belief in justice. Additionally, empathy plays a mediating role in the relationship between BJW and fair action. Thus, emphasis should be placed on nurturing nurses’ empathy skills and encouraging them to internalize principles of care and justice. Incorporating empathy training into nurse development programs and professional literacy education can foster ethical behavior, including fair conduct. Moreover, observer justice sensitivity, as a personality factor, moderates the relationship between empathy and fair behavior. These findings enable us to promote fairness among nurses by emphasizing the role of observer justice sensitivity. Nursing educators should address nurses’ sensitivity to unfair treatment of others during pre-service education, aiming to mitigate biases and unfair behaviors toward marginalized groups in their future clinical practice. As integral members of healthcare organizations, nurses should embody attributes of justice and compassion that facilitate effective fair action, a responsibility that managers should actively support.

### Limitations and recommendations for future studies

There exist some limitations to this study. First, the study design was cross-sectional, which makes it difficult to infer a causal relationship between BJW, empathy, and fair behavior. Longitudinal or experimental study designs should be employed to discover the variables’ relationship in future research. Second, the variables assessed were limited to self-reporting responses. Multiple sources of data (such as patients, physicians) should be taken to avoid common method bias as much as possible in future research. Third, the study lacks exploration into demographic variables such as economic background and social status. Future research could investigate whether these demographic variables influence nurses’ fairness behavior. Finally, this study focused only on the moderation role of observer justice sensitivity. It is yet to be established if the moderation of the perpetrator’s and observers’ and victim’ justice sensitivity is the same as the moderating effect of observer justice sensitivity on the relationship between empathy and fair behavior. Future researchers should take additional factors into account to provide a more comprehensive picture of the relationship between BJW and fair behavior.

## Conclusion

Rooting itself in the just world hypotheses, empathy-altruism hypothesis, and the protective-protective model, this study looked at the motivation of nurses’ fair behavior, specifically with regard to justice belief and caring ethics with empathy. Personality traits, emotions, and ways of thinking may all play a role in shaping the degree to which people behave selflessly toward others. The study showed that BJW was an important cognitive factor in nurses’ fair acting, and empathy is an important mediating variable through which BJW affects nurses’ fair behavior. Furthermore, observer justice sensitivity had an enhancing effect on the facilitation of fair behavior by empathy.

## Data Availability

The datasets generated and analyzed during the current study are not publicly available. The datasets are available from the corresponding author upon reasonable request.

## References

[1] Decety J, Wheatley T (2015). The Moral Brain: a multidisciplinary perspective.

[2] Contreras BP, Hoffmann AN, Slocum TA (2021). Ethical behavior analysis: evidence-based practice as a Framework for ethical decision making. Behav Anal Pract.

[3] Adamovic M (2023). Organizational justice research: a review,synthesis, and research agenda. Eur Manag Rev.

[4] Torain MJ, Bennett GG, Matsouaka RA (2021). The patient’s point of View: characterizing patient-level factors Associated with perceptions of Health Care. Heal Equity.

[5] Pérez-Arechaederra D, Briones E, Lind (2014). Perceived organizational justice in care services: creation and multi-sample validation of a measure. Soc Sci Med.

[6] Bilotta I, Dawson JF, King E (2022). B.The role of fairness perceptions in patient and employee health: a multilevel, multisource investigation. J Appl Psychol.

[7] Liang C, Gu D, Tao F (2017). Influence of mechanism of patient-accessible hospital information system implementation on doctor–patient relationships: a service fairness perspective. Inf Orm Manage-amster.

[8] Park S, Kim HK, Lee M (2023). An analytic hierarchy process analysis for reinforcing doctor–patient communication. Bmc Prim Care.

[9] De Meyer CF, Svensson G, Petzer DJ (2013). Investigating perceived justice in South African health care. Manag–J Contemp Mana.

[10] McCradden MD, Joshi S, Mazwi M (2020). Ethical limitations of algorithmic fairness solutions in health care machine learning. Lancet Digit Health.

[11] Tucker CM, Moradi B, Wall W (2014). Roles of perceived provider cultural sensitivity and health care justice in African American/Black patients’ satisfaction with provider. J Clin Psychol Med S.

[12] Zhang Y, Chen L, Xia Y. Belief in a Just World and Moral Personality as Mediating roles between parenting emotional warmth and internet altruistic behavior. Front Psychol. 2021; 1–7. 10.3389/fpsyg.2021.670373 PMC8526728 34690857

[13] Wang C, Fu W, Wu X et al. Just world beliefs and altruistic behaviors of college students during the COVID-19 pandemic: the mediating role of empathy. Curr Psychol. 2023; 1–11. 10.1007/s12144-023-04233-9 PMC9838284 36684464

[14] Feng LL, Zhong H, Zhang LL (2021). Infuence of emotional response on state empathy and helping behavior during emotion induction. China J Heal Psychol.

[15] Kutaula S, Gillani A, Leonidou LC (2022). Integrating fair trade with circular economy: personality traits, consumer engagement, and ethically-minded behavior. J bus res.

[16] Thielmann I, Spadaro G, Balliet D (2020). Personality and prosocial behavior: a theoretical framework and meta-analysis. Psychol Bull.

[17] Lerner MJ (1980). The belief in a just world: a fundamental delusion.

[18] Kiral Ucar G, Donat M, Bartholomaeus J, Thomas K, Nartova-Bochaver S (2022). Belief in a just world, perceived control, perceived risk, and hopelessness during the COVID-19 pandemic: findings from a globally diverse sample. Curr Psycho.

[19] Zheng Y, Hu D, Li X (2022). Research on the relationship between Empathy, belief in a Just World, and Childhood Trauma in Pre-clinical Medical Students. Healthcare-basel.

[20] Kiral Ucar G, Dalbert C (2020). The longitudinal associations between personal belief in a just world and teacher justice among advantaged and disadvantaged school students. Int J Psychol.

[21] Harding WG, McConatha JT, Kumar VK (2020). The relationship between Just World beliefs and life satisfaction. Int J Env Res Pub He.

[22] Thompson NM, van Reekum CM, Chakrabarti B (2022). Cognitive and affective Empathy relate differentially to emotion regulation. Affect Sci.

[23] López-Pérez B, Hanoch Y, Holt K (2017). Cognitive and affective empathy, personal belief in a just world, and bullying among offenders. J Interpers Violence.

[24] Decety J, Cowell JM (2015). Empathy, justice, and moral behavior. AJob Neurosci.

[25] Davis AN, Clark ES (2022). Considering the role of empathy in the links between discrimination and prosocial behaviors. J Adult Dev.

[26] Nook EC, Ong DC, Morelli SA (2016). Prosocial conformity: prosocial norms generalize across behavior and empathy. Pers Soc Psychol B.

[27] Miyazono K, Inarimori K (2021). Empathy Altruism, and Group Identification. Front Psychol.

[28] Fehr E, Schurtenberger I (2018). Normative foundations of human cooperation. Nat Hum Behav.

[29] Moudatsou M, Stavropoulou A, Philalithis A, Koukouli S (2020). The role of Empathy in Health and Social Care professionals. Healthc.

[30] Strauß S, Bondü R (2023). Fair sharing is just caring: links between justice sensitivity and distributive behavior in middle childhood. J Exp Child Psychol.

[31] Schlösser T, Steiniger T, Ehlebracht D (2018). How justice sensitivity predicts equality preferences in simulated democratic systems. J Res Pers.

[32] Emmanuel Köhler LJ, Strieder KL, Altenmüller MS, Gollwitzer M. Why should I? How victim sensitivity affects pro-environmental engagement. J Environ Psychol. 2024: 102276.

[33] Bondü R, Holl AK, Trommler D, Schmitt MJ (2022). Responses toward injustice shaped by Justice sensitivity - evidence from Germany. Front Psychol.

[34] Gollwitzer M, Rothmund T (2011). What exactly are victim-sensitive persons sensitive to?. J Res Pers.

[35] Bondü R, Elsner B (2015). Justice sensitivity in childhood and adolescence. Soc Dev.

[36] Chi X, Jiang W, Guo T, Hall DL, Luberto CM, Zou L. Relationship between adverse childhood experiences and anxiety symptoms among Chinese adolescents: the role of self-compassion and social support. Curr Psycho.2022; 1–13. 10.1007/s12144-021-02534-5 PMC8741560 35035184

[37] Zou Y, Wang Y, Yang X (2022). Observed ostracism and compensatory behavior: a moderated mediation model of empathy and observer justice sensitivity. Pers Indiv Differ.

[38] Pfattheicher S, Sassenrath C, Keller J (2019). Compassion magnifies third-party punishment. J Pers Soc Psychol.

[39] Memon MA, Ting H, Cheah JH, Thurasamy R, Chuah F, Cham T (2020). H.Sample size for survey research: review and recommendations. J Appl Struct Equat Model.

[40] Dalbert C (1999). The world is more just for me than generally: about the personal belief in a just world scales validity. Soc Justice Res.

[41] Vossen HG, Piotrowski JT, Valkenburg PM (2015). Development of the adolescent measure of empathy and sympathy (AMES). Pers Indiv Differ.

[42] Hong Y, Cai J, Lan R (2022). Empathy and teachers’ fairness behavior: the mediating role of moral obligation and moderating role of social value orientation. PLoS ONE.

[43] Hayes AF (2013). Introduction to mediation, moderation, and conditional process analysis. A regression-based approach.

[44] Kock F, Berbekova A, Assaf AG (2021). Understanding and managing the threat of common method bias: detection, prevention and control. Tour Manag.

[45] Harding WG, McConatha JT, Kumar VK. (2020). The Relationship between Just World Beliefs and Life Satisfaction. Int J Env Res Pub He. 2020; 17(17): 6410. 10.3390/ijerph17176410 PMC7504045 32899134

[46] O’Connell K, Brethel-Haurwitz KM, Rhoads SA, Cardinale EM, Vekaria KM, Robertson EL, Walitt B, VanMeter JW, Marsh AA (2019). Increased similarity of neural responses to experienced and empathic distress in costly altruism. Sci Rep.

[47] Messineo L, Seta L, Allegra M (2021). The relationship between empathy and altruistic motivations in nursing studies: a multi-method study. BMC Nurs.

[48] Jiang Y, Yao Y, Zhu X (2021). The infuence of college students’ empathy on prosocial behavior in the COVID-19 pandemic: the mediating role of social responsibility. Front Psychiary.

[49] Luo L, Zou R, Yang D et al. Awe experience trigged by fghting against COVID-19 promotes prosociality through increased feeling of connectedness and empathy. J Posit Psychol 2022; 866–82.

